# A Prospective Study on the Functional Outcomes of Surgical Management of Lumbar and Lumbosacral Spondylolisthesis Using Pedicle Screw Fixation and Posterolateral Fusion

**DOI:** 10.7759/cureus.83934

**Published:** 2025-05-11

**Authors:** Charan Sai Reddy Munugala, Ravikumar Biradar

**Affiliations:** 1 Orthopedics, Bijapur Liberal District Education Association (BLDE; Deemed to be University) Shri B.M. Patil Medical College, Hospital, and Research Centre, Vijayapura, IND

**Keywords:** instrumented posterolateral fusion (plf), lumbar spondylolisthesis, lumbosacral spondylolisthesis, modified oswestry disability score, pedicle screw placement, three level fixation, traumatic lumbar spondylolisthesis

## Abstract

Introduction

Low back pain is one of the most commonly reported health concerns worldwide. Its etiologies include mechanical injuries, overuse, and nerve compression resulting from various spinal disorders such as spondylolisthesis, disc herniation, spinal canal stenosis, and degenerative disc disease. Among these, spondylolisthesis - the anterior displacement of one vertebra over another - frequently contributes to spinal instability. The term is derived from the Greek words *spondylos *(vertebra) and *olisthesis *(slip). Spondylolisthesis may arise due to ligamentous laxity, pars interarticularis defects, trauma, or post-surgical procedures. In symptomatic cases, pedicle screw fixation combined with posterolateral fusion (PLF) is a well-established surgical technique aimed at stabilizing the affected spinal segments, achieving vertebral fusion, and preventing further slippage progression.

Methodology

It is a prospective observational study. The patients who met the inclusion and exclusion criteria were admitted to the Department of Orthopedics, Bijapur Liberal District Education Association (BLDE; Deemed to be University), Shri B. M. Patil Medical College, Hospital and Research Centre, Vijayapura, Karnataka, India. The patients were informed about the study in all respects, and written informed consent was obtained. The study was carried out between May 1, 2023, and December 1, 2024, with a follow-up period of six months postoperatively. Data were collected on patient demographics, symptom duration, radiographic findings, surgical parameters, and clinical outcomes.

Results

A total of 36 patients were evaluated postoperatively (16 males and 20 females). The radiological fusion rate at six months was 72.2%. The average operative time (from incision to wound closure) was 3.5 hours, and the mean intraoperative blood loss was 248 mL. Statistically significant improvements were observed in visual analog scale (VAS) scores and modified Oswestry Disability Index (ODI) at the six-month follow-up. The VAS score improvement showed a *P*-value < 0.00001, confirming its high level of significance. Functional outcomes, as measured by the modified ODI and analyzed using the Wilcoxon signed-rank test, also demonstrated substantial improvement in patient-reported quality of life.

Conclusions

Our study concludes that pedicle screw-rod instrumentation combined with PLF is a safe, effective, and reliable surgical option for the treatment of low-grade spondylolisthesis. The technique demonstrated minimal postoperative complications and provided substantial improvements in both pain relief and functional outcomes. The favorable results were observed in patients with preoperative neurological deficits, lower degrees of vertebral slip, and multi-level fusion. Given these findings, we advocate for the use of pedicle screw-rod systems with PLF as a preferred modality for managing lumbar and lumbosacral spondylolisthesis.

## Introduction

Low back pain is one of the most frequently reported health complaints worldwide, with significant socioeconomic and public health implications. The etiology is multifactorial, mainly due to injuries, repetitive strain, and nerve compression due to various disorders such as spondylolisthesis, disc herniation, spinal canal stenosis, and degenerative disc diseases.

Spondylolisthesis, a common cause of spinal instability, refers to the anterior displacement of one vertebra over another. It is derived from the Greek words *Spondylos* (vertebra) and *olisthesis* (slip) [1]. The condition was first identified by a Belgian obstetrician, Herbineaux, and Killian introduced the term. Spondylolisthesis may arise due to ligamentous laxity, pars interarticularis defects, and post-surgical procedures or may occur as a result of trauma affecting about 5% of the total population [2].

Classification plays a crucial role in the diagnosis and management of spondylolisthesis. Neugebauer and Newman categorized spondylolisthesis into five distinct types, while Meyerding’s grading system classifies spondylolisthesis based on the percentage of vertebral slippage: Grades I and II are considered low-grade, and Grades III to V are classified as high-grade spondylolisthesis. Indications for surgical intervention include neurogenic claudication, persistent radiculopathy, refractory mechanical back pain, neurological deficits, failed medical and physiotherapy, demonstrable instability, and progressing spondylolisthesis.

Significant advances in comprehending spinal biomechanics, enhancements in bone fusion methods, invention of diverse spinal instrumentation tools, advancements in surgical techniques, the evolution of MIS surgeries, etc., have enhanced the safety and efficacy of spinal fusion procedures. The primary objective of spinal fusion surgery is to alleviate pain by eliminating pathological motion and stabilizing unstable spinal segments. A variety of other surgical techniques that are currently employed for the treatment of lumbar spondylolisthesis include Pars repair, decompression, in-situ fusion, bilateral posterolateral fusion (PLF), anterior lumbar interbody fusion (ALIF), transforaminal lumbar interbody fusion (TLIF), posterior lumbar interbody fusion (PLIF), posterior instrumentation with reduction and fusion, and anterior fusion and release with posterior fusion (360-degree fusion) [3]. Each technique is selected based on patient-specific anatomical and clinical considerations, with the overarching goals of symptom resolution, restoration of spinal alignment, and stabilization.

## Materials and methods

This prospective study was conducted in the Department of Orthopedics, Bijapur Liberal District Education Association (BLDE; Deemed to be University), Shri B. M. Patil Medical College, Hospital and Research Centre, Vijayapura, Karnataka, India. The study included patients diagnosed with lumbar and lumbosacral spondylolisthesis. All participants provided written informed consent after receiving detailed information about the study protocol. The study period spanned from May 1, 2023, to December 1, 2024, with follow-up assessments conducted at one month, three months, and six months postoperatively.

Sample size calculation

As per the study conducted by Etemadifar et al. [4], considering the VAS score for back pain, the mean was taken as 9 with a standard deviation of ±1.3 and a margin of error of 0.5. The sample size was computed using the following formula:

Sample size (*n*) = (*Z* * *σ*/*d*)2

where *n* is the population size, *Z* is the *Z*-score = 2.17, *d* is the margin of error = 0.5, σ is the standard deviation =1.3, α is the level of significance = 0.03, and the estimated minimum sample size for this study was set at 32 participants.

Inclusion criteria

Patients aged 18 years and above diagnosed with spondylolisthesis who had failed conservative management, presented with neurological deficits or neurogenic claudication, and provided informed consent for surgical intervention were included in the study.

Exclusion criteria

Patients with a history of previous spinal surgeries, those with congenital spinal deformities, and individuals deemed medically unfit for surgery were excluded from the study.

Patient evaluation began with detailed history taking and a thorough clinical examination. Data were collected through patient interviews using a pre-tested questionnaire, including the visual analog scale (VAS) for pain assessment and the modified Oswestry Disability Index (ODI) for functional disability. Radiological investigations included anteroposterior and lateral X-rays of the lumbosacral spine, dynamic lateral views, and magnetic resonance imaging (MRI). Additionally, all patients underwent screening of hematological, biochemical, and serological parameters.

The surgical treatment was planned carefully, and all the procedures were conducted using a posterior midline approach over the affected segment. General anesthesia was used. Laminectomy, decompression, and reduction of the structures within the spinal canal were performed as necessary, along with pedicle screw fixation and PLF. The postoperative protocol was followed accordingly, where light occupational activities were started at two to three weeks, and moderate recreational activities started at three months. Patients were followed up for at least six months, and a radiographic assessment was performed. Postop functional outcome assessment was done using a VAS score and a modified ODI questionnaire at the end of six months.

The data obtained were entered in a Microsoft Excel sheet, and statistical analyses were performed using SPSS, version 20 (IBM Corp., Armonk, NY). Descriptive statistics were calculated for all variables, including means, standard deviations, medians, and ranges for continuous data, and frequencies and percentages for categorical data. Continuous variables were expressed as means ± standard deviations, while categorical variables were presented as frequencies and percentages. Categorical variables were analyzed using the chi-square test. The Shapiro-Wilk test was used to assess the normality of data. Since the data did not follow a normal distribution, the Wilcoxon signed-rank test was applied to compare preoperative and postoperative continuous variables. Statistical significance was set at *P* < 0.05.

## Results

A total of 40 patients underwent surgical management with pedicle screw fixation and PLF for spondylolisthesis. Of these, 36 patients completed follow-up evaluations up to six months postoperatively, while four patients were lost to follow-up. Among the 36, 16 (44.4%) were male and 20 (55.6%) were female (Table 1).

**Table 1:** Distribution of patients according to gender. The data are represented as the frequency of lumbar and lumbosacral spondylolisthesis for different genders and the percentage of the population (*n*, %).

Gender	Number of patients	Percentage
Females	20	55.6%
Males	16	44.4%
Total	36	100%

Table 1 shows the gender distribution of the study, with a higher prevalence of spondylolisthesis in females (20 patients, 55.6%) compared to males (16 patients, 44.4%).

In this study, a total of 36 patients were assessed for the grade of spondylolisthesis. According to Meyerding's classification, the majority of patients (*n* = 18, 50%) had Grade I spondylolisthesis, followed by Grade II in 14 patients (38.9%) and Grade III in 4 patients (11.1%) (Table 2).

**Table 2:** Distribution of patients according to Meyerding's grade. The data are represented as the frequency of lumbar and lumbosacral spondylolisthesis categorized according to Meyerding's grade and the percentage of the population (*n*, %).

Meyerding's type	Number of patients	Percentage
Grade I	18	50%
Grade II	14	38.9%
Grade III	04	11.1%
Grade IV	0	-
Grade V	0	-

The duration of symptoms before surgery ranged from one month to eight years. All 36 patients presented with backache, while 23 (63.9%) reported with radiculopathy, and 5 patients (13.9%) exhibited neurological deficits (Table 3).

**Table 3:** Distribution of patients according clinical features. Data are represented as the frequency of symptoms in lumbar and lumbosacral spondylolisthesis.

Symptoms	Number of patients
Low backache	36
Radiculopathy	23
Deficiencies	5

Table 3 outlines the most common presenting features in the study. Low backache was present in all patients (*n* = 36), followed by radiculopathy in 23 patients (63.9%) and neurological deficiencies in 5 patients (13.9%).

The radiological union rate at six months postoperatively was 72.2%. The mean duration of surgery, measured from incision to closure, was 3.5 hours. The average intraoperative blood loss was 248 mL. A statistically significant improvement was observed in VAS scores and modified ODI score at six-month follow-up (Table 4).

**Table 4:** VAS and modified ODI score analysis. The table presents the results of a Wilcoxon signed-rank test comparing pre- and post-intervention outcomes for two variables. VAS, visual analog scale; ODI, Oswestry Disability Index

Variables	Pre		Post		Wilcoxon signed-rank test	*P*-value
	Mean	±SD	Mean	±SD		
VAS	6.3611	0.83333	1.5277	1.5210	-5.292	<0.00001
Modified ODI	59.3888	6.5695	11.2777	7.7408	-5.2316	<0.00001

Furthermore, assessment of quality of life, using the Wilcoxon signed-rank test, revealed a significant decrease in the modified ODI scores when comparing preoperative and postoperative values. These findings supported the effectiveness of pedicle screw fixation with PLF in improving both pain and functional outcomes in patients with spondylolisthesis.

VAS scores

The mean preoperative VAS score was 6.36 (SD = 0.83), while the mean postoperative VAS score was 1.52 (SD = 1.52). This difference was statistically significant (*P* < 0.00001).

Modified ODI scores

The mean preoperative ODI score was 59.38 (SD = 6.57), and the mean postoperative ODI score was 11.27 (SD = 7.74). This difference was also statistically significant (*P* < 0.00001).

The overall outcome of the study was evaluated using a composite scoring system based on VAS, modified ODI, and improvement in radiculopathy and neurological deficits (Table 5), with a maximum total of 12 points and a minimum of 4 points.

**Table 5:** Clinical scoring criteria for outcome assessment. This table outlines the clinical scoring system used to assess postoperative outcomes. Each parameter - VAS difference, modified ODI difference, radiculopathy status, and neurological deficit improvement - is assigned a score from 1 to 3 based on the degree of improvement observed after surgery. VAS, visual analog scale; ODI, Oswestry Disability Index

Points	3	2	1
VAS difference	>3.5	>3	<2.94
ODI difference	>40 %	10%-40%	<10%
Radiculopathy	Absent	Present	Persisting
Deficits	2 grades	1 grade	Not improved

Based on the cumulative scores, outcomes were categorized as excellent (score ≥10), good (score 7-9), and fair (score ≤6). In the present study, 27 patients (75%) achieved excellent outcomes, while 9 patients (25%) had good outcomes. No patients were classified in the fair outcome category (Table 6).

**Table 6:** Distribution of patient outcomes based on score. Data are represented as the frequency of lumbar and lumbosacral spondylolisthesis categorized into score ranges, along with the number of patients and percentage of the population (score range, n, %). These reflect clinical outcomes, with higher scores indicating better outcomes.

Outcome	Score range	Number of patients	Percentage
Excellent	≥10	27	75.0
Good	7-9	9	25.0
Fair	≤6	0	0.0
Total	-	36	100.0

Outcomes did not demonstrate a statistically significant difference based on gender (Table 7). However, younger patients exhibited more favorable outcomes, which may be attributed to better bone quality, enhanced healing capacity, and better overall recovery potential (Table 8). Additionally, patients diagnosed with low-grade spondylolisthesis showed more positive postoperative outcomes compared to those with higher-grade lesions (Table 9).

**Table 7:** Postoperative outcomes based on gender. This table presents the distribution of postoperative clinical outcomes based on patient sex. Outcomes were categorized as excellent, good, and fair.

Sex	Number of patients	Excellent	Good	Fair
Male	16	12	4	0
Female	20	15	5	0

**Table 8:** Postoperative outcomes based on age. This table presents the distribution of postoperative clinical outcomes across different age groups. Outcomes were categorized as excellent, good, or fair based on a standardized scoring system.

Age group	Number of patients	Excellent	Good	Fair
<40	9	9	0	0
41-50	9	9	0	0
51-60	13	7	6	0
>60	5	2	3	0

**Table 9:** Distribution of outcomes by Meyerding's grade. This table presents the number of patients and their outcomes, categorized by Meyerding's grade of spondylolisthesis. Outcomes are defined as follows: Excellent (score ≥ 10), Good (score 7–9), and Fair (score ≤ 6).

Meyerding's grade	Number of patients	Excellent	Good	Fair
I	18	16	2	0
II	14	9	5	0
III	4	2	2	0
IV	0	0	0	0
V	0	0	0	0

Postoperative outcomes were compared between male and female patients to assess any association between gender and clinical improvement. Among the 36 patients, 12 out of 16 males (75%) achieved excellent outcomes, and 4 (25%) had good outcomes. Among 20 female patients, 15 (75%) had excellent results and 5 (25%) had good results. No fair outcomes were observed in either group. A chi-square test showed no statistically significant association between sex and clinical outcome (*χ*² = 0.00, *P* = 1.00), indicating that outcomes were comparable between male and female patients.

Clinical outcomes varied across different age groups. In patients under 40 years (*n* = 9) and those aged 41-50 years (*n* = 9), all achieved excellent results (100%). Among patients aged 51-60 years (*n* = 13), 7 (53.8%) had excellent outcomes and 6 (46.2%) had good outcomes. In the >60 age group (*n* = 5), 2 (40%) had excellent results and 3 (60%) had good outcomes. No fair outcomes were observed in any age group. A chi-square test demonstrated a statistically significant association between age group and outcome (*χ*² = 12.37, *P *= 0.0062), suggesting that younger patients had better clinical results compared to older individuals.

According to Meyerding’s grading of spondylolisthesis, excellent outcomes were predominantly seen in patients with lower grades. Among 18 patients with Grade I, 16 (88.9%) had excellent outcomes and 2 (11.1%) had good outcomes. In Grade II (*n* = 14), 9 patients (64.3%) achieved excellent results , and 5 (35.7%) had good outcomes. For Grade III (*n* = 4), 2 patients (50%) had excellent and 2 (50%) had good results. However, the chi-square test (*χ*² = 4.04, *P* = 0.13) indicates no statistically significant association, though a trend toward better outcomes with lower-grade slips was observed.

Although existing literature advocates for fusing the minimal number of spinal segments necessary to achieve stability, findings from our study suggest that three-level fusion resulted in superior functional outcomes and greater pain relief compared to two-level fusion. This observation may reflect improved stabilization and decompression over a broader region, contributing to better overall clinical improvement (Table 10).

**Table 10:** Distribution of clinical outcomes by number of levels fixed. This table presents data on patients and their outcomes, categorized by the number of levels fixed. Outcomes are defined as follows: Excellent (score ≥ 10), Good (score 7–9), and Fair (score ≤ 6).

Fixation level	Number of patients	Excellent	Good	Fair
Two-level	24	16	8	0
Three-level	12	11	1	0

Excellent results were observed in 11 out of 12 patients (91.6%) who underwent three-level fixation, compared to 16 out of 24 patients (66.6%) who received two-level fixation. However, this difference was not statistically significant with the chi-square test (χ² = 1.5, *P* = 0.22).

Adult spondylolisthesis is typically diagnosed through radiographic assessment of lumbar segment instability and vertebral slippage. Early and accurate identification of the distinct clinical features, neurological signs, and functional limitations is crucial in differentiating spondylolisthesis from other causes of low back pain and sciatica. Despite undergoing conservative treatment, a significant proportion of patients continue to experience persistent symptoms, highlighting the need for appropriate surgical intervention in carefully selected cases.

Case illustration

**Figure 1:**
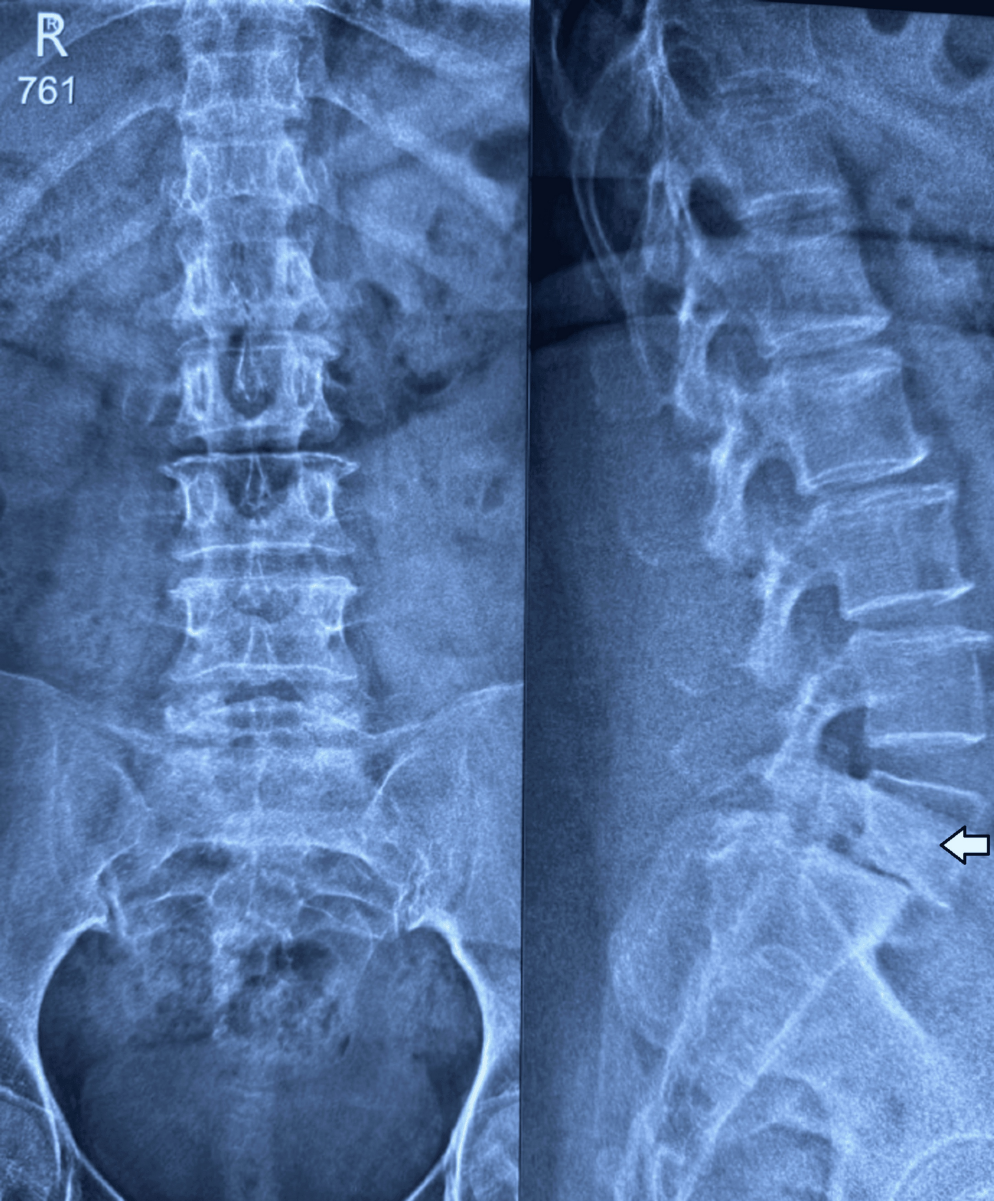
X-ray of the lumbosacral spine: anteroposterior and lateral views. Plain radiograph of a skeletally mature patient showing L5-S1 Meyerding’s Grade II spondylolisthesis (anterolisthesis).

**Figure 2:**
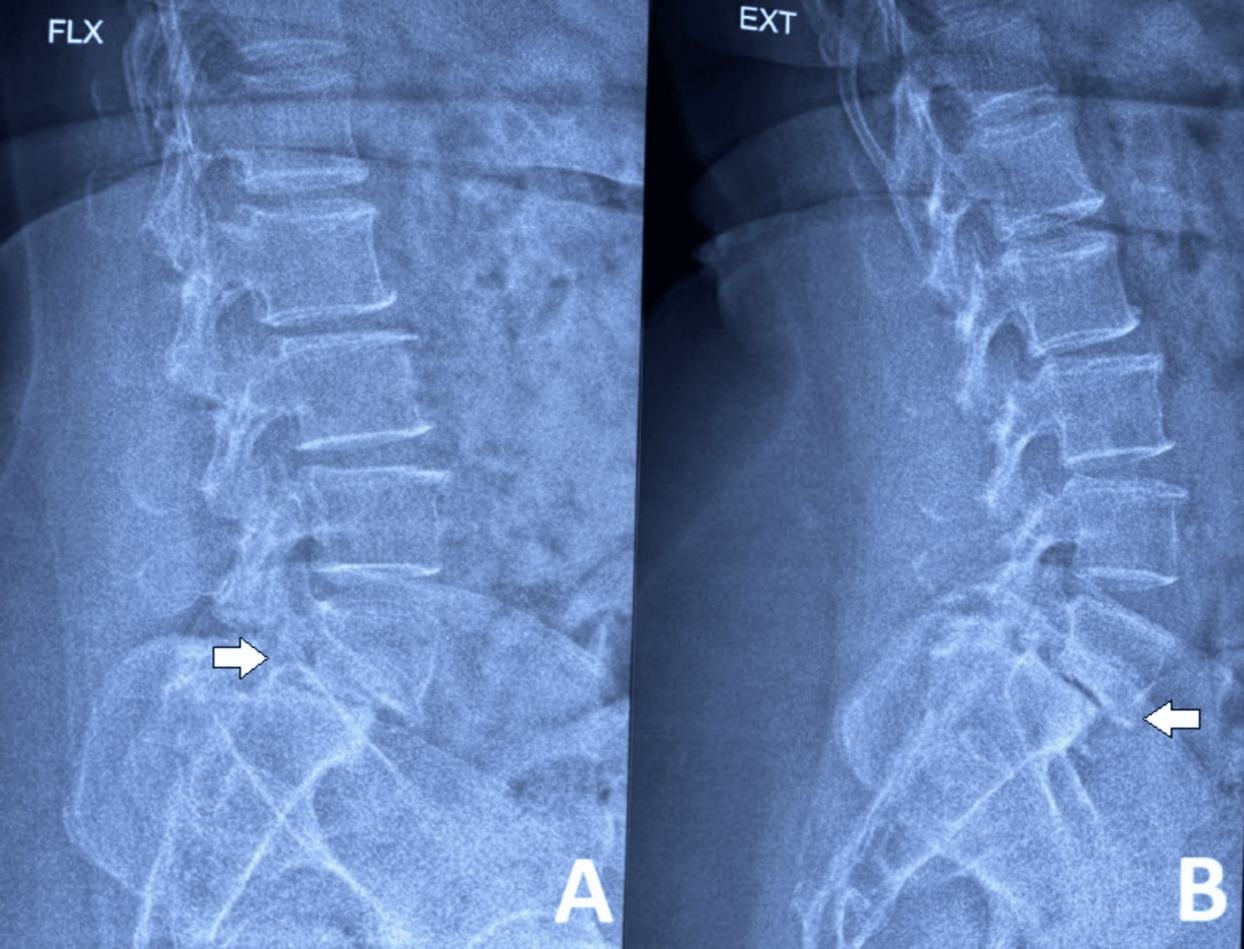
X-ray of the lumbosacral spine: dynamic view Plain radiograph of the lumbosacral spine in lateral flexion (A) and extension (B) views, showing instability with notable end plate sclerosis at L5 and S1.

**Figure 3:**
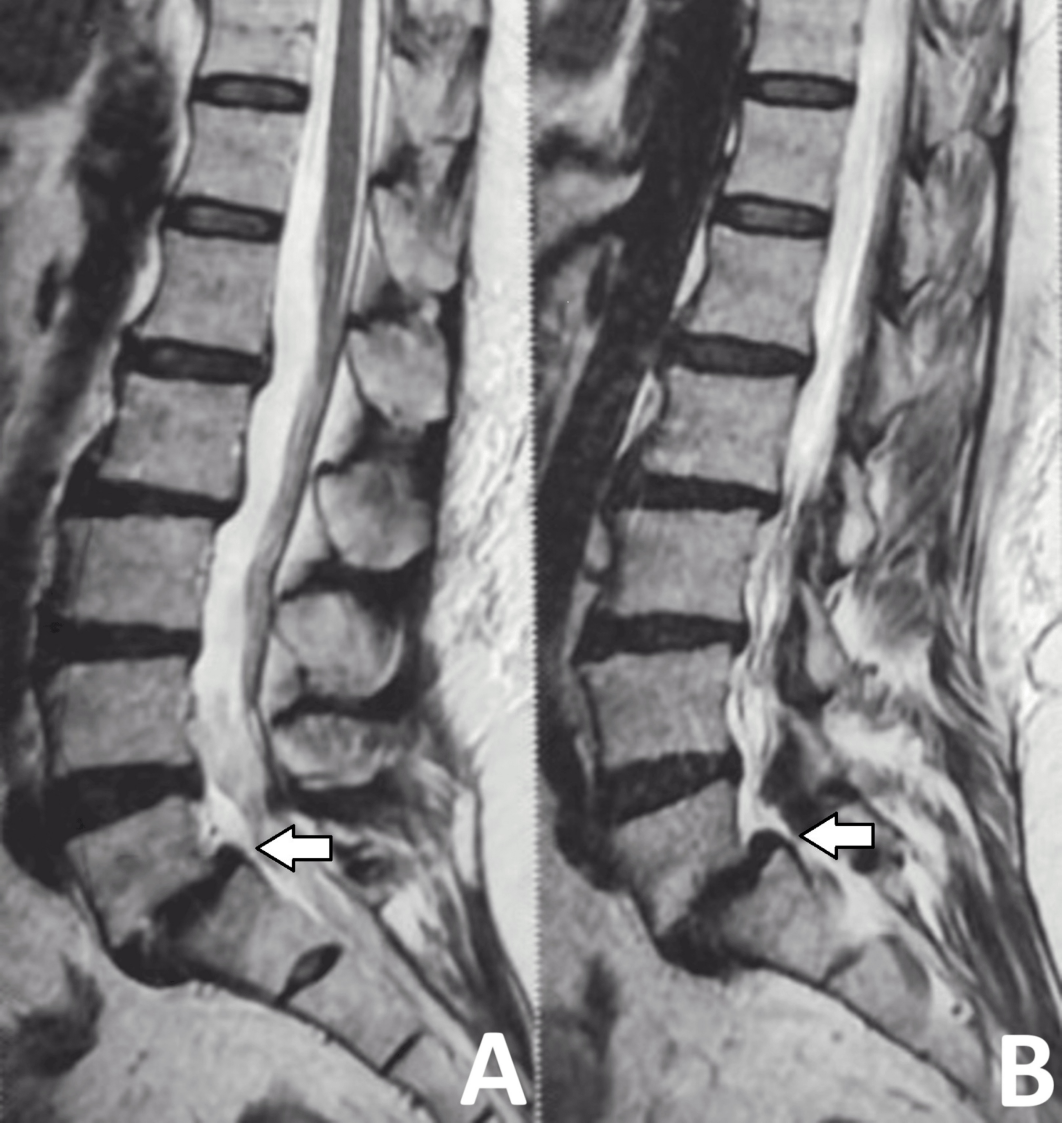
MRI of the lumbosacral spine in sagittal view. T2-weighted MRI of the lumbosacral spine showing L5-S1 spondylolisthesis with a pseudo disc bulge, labeled in two different sagittal sections (A and B).

**Figure 4:**
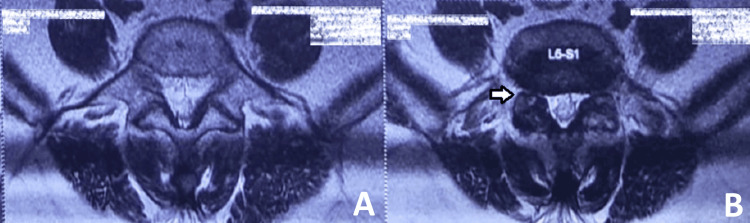
MRI of the lumbosacral spine in axial view. T2-weighted MRI of the lumbosacral spine in axial view at the bone (A) and disc level (B), showing foraminal stenosis due to a pseudo-disc bulge at the L5-S1 level (labeled in B).

**Figure 5:**
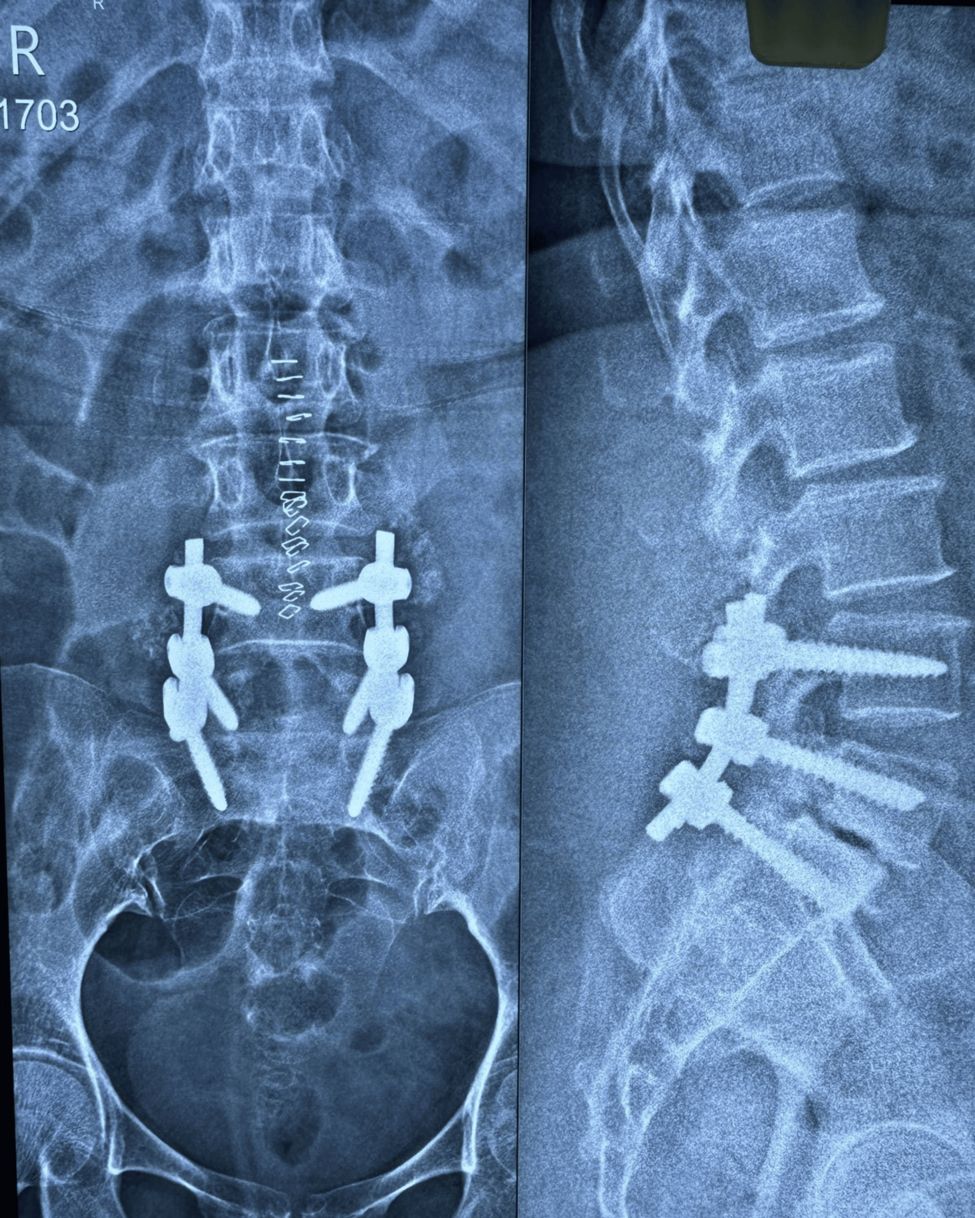
Immediate postoperative X-ray of the lumbosacral spine, showing anteroposterior and lateral views. Plain radiography of the lumbosacral spine showing anteroposterior and lateral views with three-level fixation of L5-S1 spondylolisthesis, including L4-L5 and L5-S1 pedicle screw fixation and posterolateral fusion.

**Figure 6:**
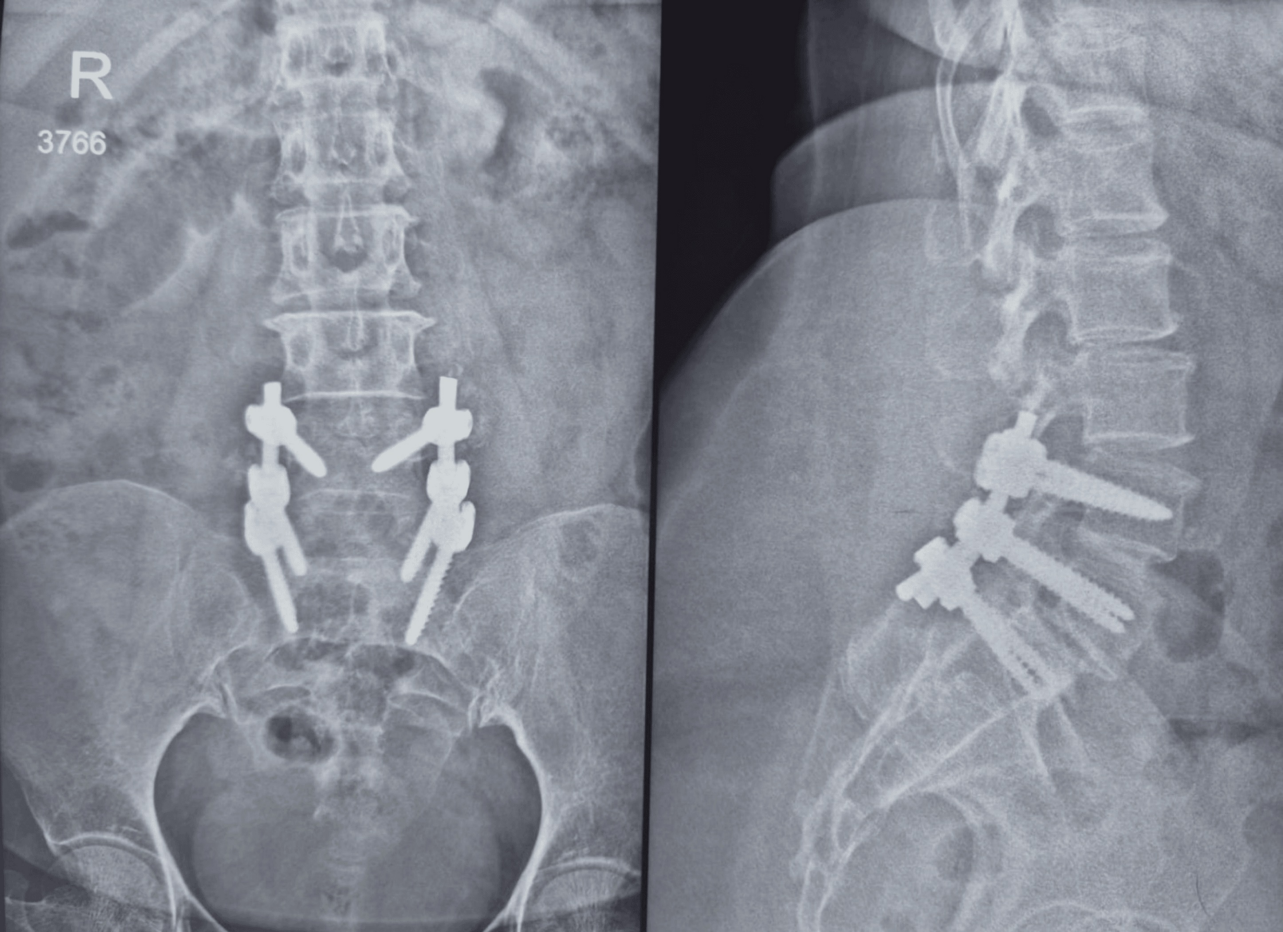
Six-month postoperative X-ray of the lumbosacral spine, showing anteroposterior and lateral views. Plain radiography of the lumbosacral spine showing anteroposterior and lateral views with three-level fixation of L5-S1 spondylolisthesis, including L4-L5 and L5-S1 pedicle screw fixation and posterolateral fusion, six months postoperatively, showing features of fusion.

A 46-year-old female patient was diagnosed with L5 over S1 anterolisthesis. She presented with complaints of low back pain and radiculopathy radiating to the right lower limb for the past nine months. Figures 1-6 depict the preoperative radiographs and MRI scans, as well as immediate and six-month postoperative radiographs.

## Discussion

The primary objective in the surgical management of lumbar and lumbosacral spondylolisthesis is to achieve solid bony fusion. Fusion rates generally improve with longer follow-up durations, regardless of the type of instrumentation used. Despite the limited sample size and relatively short follow-up period in our study, the radiological union rate of 72.2% at six months is consistent with fusion rates reported in the literature over similar intervals. Notably, despite the partial radiological union, clinical outcomes demonstrated significant functional improvement, as evidenced by substantial reductions in VAS and ODI scores.

In one study, the mean age of patients undergoing PLF was 47.3 years [5]. In our cohort, the mean age was slightly higher, at 49.7 years, with a predominance of female patients. The duration of symptoms ranged from 1 month to 8 years, with a mean duration of 26.4 months before surgical intervention. 

The most frequently involved spinal segment in our series was L4-L5, accounting for 20 cases (55.6%), followed by L5-S1 in 15 cases (41.7%) and L3-L4 in one case (2.7%). These findings are comparable to those reported by Kim et al., who observed 50% involvement of L4-L5 [6]. Similarly, Dantas et al. reported an equal distribution (45%) between L4-L5 and L5-S1 levels [7], while Yan et al. noted 52.27% involvement at L5-S1 and 47.72% at L4-L5 [8]. Concerning Meyerding’s classification, our study identified Grade I spondylolisthesis in 18 patients (50%), Grade II in 14 patients (38.9%), and Grade III in 4 patients (11.1%), highlighting the predominance of low-grade slips in our study.

PLF has been established as a standard surgical approach for treating lumbar spinal instability. With the advancements in spinal instrumentation techniques, this procedure continued to be in practice for degenerative spondylolisthesis. Of the 36 patients examined, 26 (72.2%) successfully attained bony fusion, while 10 did not. The average duration to achieve bony fusion was 5.5 months. Favorable outcomes were notably associated with younger patients, lower levels of slippage, and individuals in the radiological fusion category. These findings underscore the importance of accounting for spinal biomechanics in the surgical treatment of spondylolisthesis to optimize outcomes [9].

Crawford et al. explored the biomechanics of various hardware combinations in a cadaveric model of Grade I spondylolisthesis, comparing pedicle screws, cages with or without intersomatic spacers, and their combinations. Their study emphasized the mechanical impact of instrumentation choices on stability [10]. Despite these insights, consensus is lacking on the optimal surgical technique, as the preferred approach must strike a balance between minimizing the number of fused segments, achieving effective decompression, maintaining sagittal alignment, and ensuring fusion success [11].

Suk et al. conducted decompression, pedicle screw fixation, and fusion in 76 patients experiencing symptomatic spondylolisthesis along with a stenotic spinal canal. In the PLF group, the nonunion rate was 7.5%, while the PLIF group had no instances of nonunion [12]. Nevertheless, a study could not definitively determine which surgical technique (PLF, PLIF, ALIF, or instrumentation) was the most effective for achieving fusion. In treating low-grade isthmic spondylolisthesis, the roles of instrumentation, decompression, reduction, and fusion can all be beneficial [13].

A prospective study by Kim et al. comparing PLF, PLIF, and combined PLF/PLIF approaches over three years reported no statistically significant differences in fusion rates or clinical outcomes. However, the PLIF-only group had advantages in terms of shorter operative times, reduced intraoperative blood loss, and absence of iliac crest donor site pain, often encountered in PLF procedures.

In a comparative analysis by Madan and Boeree, clinical outcomes were satisfactory in 69.5% of both PLIF and PLF groups. While PLF yielded better clinical outcomes in low-grade cases, PLIF was superior in terms of radiological fusion quality and slip correction, highlighting the technique-specific advantages and the importance of individualized surgical planning [14].

Ekman et al., after two years of experience with both PLF and PLIF, concluded that both techniques yielded comparable clinical outcomes in the treatment of adult isthmic spondylolisthesis, though PLIF was associated with a higher rate of complications [15]. Their findings emphasized that adequate fusion strongly correlated with better clinical outcomes, whereas failure to achieve fusion was frequently linked with suboptimal results. Several studies have similarly reported that PLIF does not consistently outperform other fusion techniques in terms of functional recovery. Notably, the extensive nerve root and thecal sac retraction required during PLIF procedures has been identified as a disadvantage, with some patients experiencing postoperative leg pain as a result.

In a comparative analysis, the ODI showed that 89% of patients who underwent PLIF and 86% of those who underwent PLF reported good or excellent outcomes, although the difference was not statistically significant [16,17]. A prospective randomized trial evaluating PLF, PLIF, and a combined PLF+PLIF approach in patients with degenerative lumbar disease also found no significant differences in either clinical outcomes or fusion rates among the three groups [18].

Complications associated with PLF include implant-related issues such as peri-implant fractures and hardware loosening, which may necessitate reoperation. It is generally accepted that surgical reduction is not required in cases of symptomatic Grade I or II spondylolisthesis, as patients who underwent reduction procedures were found to have a higher complication rate. Although successful radiological fusion is often considered a key marker of surgical success in mechanical low back pain, several studies have failed to establish a direct correlation between fusion status and clinical outcome [19,20].

A prospective study aimed to evaluate and compare the clinical outcomes of PLIF and PLF in cases of spondylolisthesis. Radiographs were conducted both before and after surgery to assess fusion status. Both surgical techniques proved effective; however, the PLF group exhibited a higher incidence of complications related to hardware mechanics. The PLIF group achieved a superior fusion rate compared to the PLF group, yet there was no significant statistical variation in clinical and functional outcomes between the two groups [21,22].

Swan et al. compared two cohorts of patients with low-grade isthmic spondylolisthesis, wherein one group (*n* = 50) underwent posterior instrumentation with PLF, and the other group (*n *= 50) received a combined ALIF and PLF. At the two-year follow-up, the combined anterior and posterior approach demonstrated superior correction of vertebral slippage compared to the posterior-only technique [23].

Spinal procedures are not without complications. Permanent neurological deficits occur in 0.4%-1.7%, cerebrospinal fluid (CSF) leaks in 0.4%-0.5%, radicular pain in 1.1%-2.5%, and deep wound infections in 0.6%-5% of cases [24]. The complication rates associated with PLIF are generally higher than those seen with PLF. From a technical perspective, PLF is simpler to perform, involves less intraoperative blood loss, and avoids some of the anatomical challenges of PLIF, which requires more extensive dissection, longer operative time, and carries a greater risk of CSF leaks [25]. These leaks, which may arise from surgical trauma, can lead to persistent headaches and, in rare cases, meningitis, necessitating surgical repair involving direct dural closure or fascial grafting.

One case of intraoperative dural injury was encountered, which was managed using a muscle graft. The patient later developed a soft swelling over the surgical site, which resolved spontaneously over three months. Additionally, one patient developed a postoperative wound infection, which was effectively managed with intravenous antibiotics administered according to the culture and sensitivity report.

Surgical intervention is frequently required and necessitates careful direct closure of the dura or closure using a fascial graft. Based on the findings of one study, it was determined that in spondylolisthesis cases involving three-column spinal instability, PLIF using pedicle screws provides a stronger mechanical framework than pedicle screw fixation alone. While both surgical techniques were adequate, the PLF group was associated with more complications related to hardware mechanics [26]. When comparing cost-effectiveness, PLF demonstrates superiority over other fusion techniques due to its relative simplicity and shorter operative time, which contribute to reduced overall healthcare costs.

Clinical and functional outcomes were comparable in both groups; no significant statistical differences were observed. However, PLIF demonstrated a higher fusion rate relative to PLF. Conservative treatment alternatives for segmental instability are suitable for patients experiencing manageable pain [27]. Surgical options become necessary when symptoms are severe enough to disrupt daily activities, if the condition worsens, or if there are notable neurological impairments. PLF can be an effective treatment option for lumbar spondylolisthesis management [28].

This study is not without limitations. The relatively small sample size is partly attributable to the tier-2 setting of our institution in North Karnataka, India, where a significant proportion of patients often seek care in metropolitan centers and have a shorter follow-up time, as the majority of patients are not in favor of longer follow-ups in the absence of symptoms. Nevertheless, our findings are consistent with existing literature, reinforcing the efficacy and safety of pedicle screw fixation with PLF in low-grade spondylolisthesis. Future studies incorporating larger, multicentric cohorts are essential. Moreover, advances in minimally invasive spine surgery (MISS) and the use of intraoperative microscopy offer opportunities to compare PLF with newer techniques, particularly in cases of degenerative spondylolisthesis, with respect to achieving adequate decompression, circumferential fusion, and minimizing surgical morbidity.

## Conclusions

Spondylolisthesis is a prevalent condition frequently encountered in orthopedic practices related to low backache and radiculopathy. Surgical decompression and stabilization of the spine are advised for patients who do not improve with conservative treatments or have significant spinal instability. Pedicle screw fixation with PLF is a safe and effective option with minimal complications, particularly for low-grade spondylolisthesis.

Our findings indicate that pedicle screw fixation with PLF and decompression was the primary surgical approach employed for the treatment of spondylolisthesis at our institution. Postoperative complications were minimal, particularly when procedures were performed under fluoroscopic guidance. Favorable outcomes were predominantly associated with the presence of preoperative neurological deficits, the degree of vertebral slip, and the number of spinal segments fused. Alongside surgical intervention, lifestyle modifications are recommended to reduce the risk of surgical failure and enhance long-term outcomes. Although preliminary evidence from existing studies supports the effectiveness of this approach, the limited sample sizes underscore the need for further research involving larger patient cohorts to validate these findings and explore alternative surgical techniques such as PLIF, TLIF, and ALIF, particularly for high-grade spondylolisthesis.

## References

[1] Azar FM, Beaty JH (2021). Campbell’s Operative Orthopaedics.

[2] Chandler Chandler, Fremont A (1931). Lesions of the “isthmus”(pars interarticularis) of the laminae of the lower lumbar vertebrae and their relation to spondylolisthesis. Surg Gynecol Obstet.

[3] Vibert BT, Sliva CD, Herkowitz HN (2006). Treatment of instability and spondylolisthesis: surgical versus nonsurgical treatment. Clin Orthop Relat Res.

[4] Etemadifar MR, Hadi A, Masouleh MF (2016). Posterolateral instrumented fusion with and without transforaminal lumbar interbody fusion for the treatment of adult isthmic spondylolisthesis: a randomized clinical trial with 2-year follow-up. J Craniovertebr Junction Spine.

[5] Jacobs WC, Vreeling A, De Kleuver M (2006). Fusion for low-grade adult isthmic spondylolisthesis: a systematic review of the literature. Eur Spine J.

[6] Kim JS, Lee KY, Lee SH, Lee HY (2010). Which lumbar interbody fusion technique is better in terms of level for the treatment of unstable isthmic spondylolisthesis?. J Neurosurg Spine.

[7] Dantas FL, Prandini MN, Ferreira MA (2007). Comparison between posterior lumbar fusion with pedicle screws and posterior lumbar interbody fusion with pedicle screws in adult spondylolisthesis. Arq Neuropsiquiatr.

[8] Yan DL, Pei FX, Li J, Soo CL (2008). Comparative study of PILF and TLIF treatment in adult degenerative spondylolisthesis. Eur Spine J.

[9] Chaitanya M, Mittal A, Rallapalli R (2015). Surgical management of spondylolisthesis by pedicular screw rod system and postero-lateral fusion. Open J Orthop.

[10] Crawford NR, Cagli S, Sonntag VK, Dickman CA (2001). Biomechanics of grade I degenerative lumbar spondylolisthesis. Part 1: in vitro model. J Neurosurg.

[11] Inamdar DN, Alagappan M, Shyam L, Devadoss S, Devadoss A (2006). Posterior lumbar interbody fusion versus intertransverse fusion in the treatment of lumbar spondylolisthesis. J Orthop Surg (Hong Kong).

[12] Suk SI, Lee CK, Kim WJ, Lee JH, Cho KJ, Kim HG (1997). Adding posterior lumbar interbody fusion to pedicle screw fixation and posterolateral fusion after decompression in spondylolytic spondylolisthesis. Spine (Phila Pa 1976).

[13] Kant AP, Daum WJ, Dean SM (1995). Evaluation of lumbar spine fusion: plain radiographs versus direct surgical exploration and observation. Spine.

[14] Madan S, Boeree NR (2002). Outcome of posterior lumbar interbody fusion versus posterolateral fusion for spondylolytic spondylolisthesis. Spine (Phila Pa 1976).

[15] Ekman P, Möller H, Tullberg T, Neumann P, Hedlund R (2007). Posterior lumbar interbody fusion versus posterolateral fusion in adult isthmic spondylolisthesis. Spine (Phila Pa 1976).

[16] Hacker RJ (1997). Comparison of interbody fusion approaches for disabling low back pain. Spine (Phila Pa 1976).

[17] Wetzel FT, LaRocca H (1991). The failed posterior lumbar interbody fusion. Spine (Phila Pa 1976).

[18] Dickman CA, Fessler RG, MacMillan M, Haid RW (1992). Transpedicular screw-rod fixation of the lumbar spine: operative technique and outcome in 104 cases. J Neurosurg.

[19] Naderi S, Manisali M, Acar F, Ozaksoy D, Mertol T, Arda MN (2003). Factors affecting reduction in low-grade lumbosacral spondylolisthesis. J Neurosurg.

[20] Poussa M, Schlenzka D, Seitsalo S (1993). Surgical treatment of severe isthmic spondylolisthesis in adolescents: reduction or fusion in situ. Spine.

[21] Turner JA, Ersek M, Herron L, Haselkorn J, Kent D, Ciol MA, Deyo R (1992). Patient outcomes after lumbar spinal fusions. JAMA.

[22] Cheng L, Nie L, Zhang L (2009). Posterior lumbar interbody fusion versus posterolateral fusion in spondylolisthesis: a prospective controlled study in the Han nationality. Int Orthop.

[23] Swan J, Hurwitz E, Malek F, van den Haak E, Cheng I, Alamin T, Carragee E (2006). Surgical treatment for unstable low-grade isthmic spondylolisthesis in adults: a prospective controlled study of posterior instrumented fusion compared with combined anterior-posterior fusion. Spine J.

[24] Matthiass HH, Heine J (1986). The surgical reduction of spondylolisthesis. Clin Orthop Relat Res.

[25] Spruit M, Pavlov PW, Leitao J, De Kleuver M, Anderson PG, Den Boer F (2002). Posterior reduction and anterior lumbar interbody fusion in symptomatic low-grade adult isthmic spondylolisthesis: short-term radiological and functional outcome. Eur Spine J.

[26] Boriani S, Weinstein JN, Biagini R (1997). Primary bone tumors of the spine. Terminology and surgical staging. Spine (Phila Pa 1976).

[27] Brantigan JW, Neidre A (2003). Achievement of normal sagittal plane alignment using a wedged carbon fiber reinforced polymer fusion cage in treatment of spondylolisthesis. Spine J.

[28] Benguluri R, Kumar CS (2018). Surgical management of spondylolysthesis by pedicular screw rod system and postero-lateral fusion. IOSR J Dent Med Sci.

